# Mesoporous Silica Xerogels Prepared by *p*-toluenesulfonic Acid-Assisted Synthesis: Piperazine-Modification and CO_2_ Adsorption

**DOI:** 10.3390/nano15191459

**Published:** 2025-09-23

**Authors:** Stela Grozdanova, Ivalina Trendafilova, Agnes Szegedi, Pavletta Shestakova, Yavor Mitrev, Ivailo Slavchev, Svilen Simeonov, Margarita Popova

**Affiliations:** 1 Institute of Organic Chemistry with Centre of Phytochemistry, Bulgarian Academy of Sciences, 1113 Sofia, Bulgaria; stela.grozdanova@orgchm.bas.bg (S.G.); ivalina.trendafilova@orgchm.bas.bg (I.T.); pavletta.shestakova@orgchm.bas.bg (P.S.); yavor.mitrev@orgchm.bas.bg (Y.M.); ivailo.slavchev@orgchm.bas.bg (I.S.); svilen.simeonov@orgchm.bas.bg (S.S.); 2 National Centre of Excellence Mechatronics and Clean Technologies, 8 bul. Kliment Ohridski, 1113 Sofia, Bulgaria; 3 Institute of Materials and Environmental Chemistry, HUN-REN Research Centre for Natural Sciences, Magyar tudósok krt. 2, 1117 Budapest, Hungary; szegedi.agnes@ttk.hu

**Keywords:** mesoporous silica, organic acid assisted synthesis, surface modification, piperazine, CO_2_ adsorption

## Abstract

*p*-toluenesulfonic acid (pTSA) was used for the synthesis of porous silica xerogels while applying different synthesis conditions. Key parameters included acid concentration, drying temperature and the method of acid removal. The resulting organic–inorganic composites were investigated by nitrogen physisorption, X-ray powder diffraction (XRD), solid-state NMR and thermal analysis. The results demonstrated that both the drying temperature and quantity of the pTSA significantly influenced the pore structure of the xerogels. The utilization of such strong acids like pTSA yielded high surface area and pore volume, as well as narrow pore size distribution. Environmentally friendly template removal by solvent extraction produced materials with superior textural properties compared to traditional calcination, enabling the recovery and reuse of pTSA with over 95% efficiency. A selected mesoporous silica xerogel was modified by a simple two-step post-synthesis procedure with 1-(2-Hydroxyethyl) piperazine (HEP). High CO_2_ adsorption capacity was determined for the HEP-modified material in dynamic conditions. The isosteric heat of adsorption revealed the stronger interaction between functional groups and CO_2_ molecules. Total CO_2_ desorption could be achieved at 60 °C. Leaching of the silica functional groups could not be detected even after four consecutive adsorption cycles. These findings provide valuable insights into the sustainable synthesis of tunable piperazine-modified mesoporous silica xerogels with potential applications in CO_2_ capture.

## 1. Introduction

The continuous rise in atmospheric carbon dioxide (CO_2_) levels driven largely by fossil fuel combustion has emerged as a central concern in the context of global climate change. Effective capture and separation of CO_2_ from point sources such as flue gas is a critical strategy to mitigate greenhouse gas emissions [1,2]. There are three main technologies by which carbon capture can be implemented—precombustion, capture during combustion, and postcombustoin [3]. The postcombustion method is favored more since it does not require the modification of existing facilities in order to function. To this day, the main postcombustoin CO_2_ separation process involves the use of amines such as monoethanolamine (MEA), diethanolamine (DEA), and methyldiethanolamine (MDEA) in solution. However, some of their major drawbacks are that they are corrosive [4], and during operation there is a loss of amines and they need a lot of energy for their regeneration [5]. Among the various capture technologies, adsorption-based methods are particularly attractive due to their relatively low energy requirements, scalability, and potential for integration with existing industrial systems [6]. Some of the main criteria for selecting CO_2_ sorbent materials are their CO_2_ adsorption capacity, CO_2_ selectivity, the kinetics for CO_2_ adsorption and desorption, and the stability of the materials [7]. Mesoporous silica materials have attracted significant interest as supports for CO_2_ adsorbents because of their high surface area, uniform mesoporosity, and excellent thermal and mechanical stability [8,9]. These materials are typically synthesized using surfactants which self-assemble into ordered structures while the silica condenses around them. The surfactants are then removed and leave mesoporous ordered composites [10]. The ordered pore structures (e.g., SBA-15, MCM-41) not only facilitate rapid mass transfer but also provide an ideal platform for surface functionalization. Over the years new methods have been developed for the synthesis of such materials [11]. In some of them, organic acids are used as templates for the formation of mesoporous silica [12,13]. However, their synthesis involves the use of an additional acid to hydrolyse the silica source before interacting with the template. Another paper shows how citric acid could be used both as a template and as a catalyst for hydrolysis [14]. This method is time-consuming and the extraction of the template is carried out with ethanol, which is not economical. In the early 90s, Coltrain et al. [15] used different acids as catalysts for the synthesis of silica materials. They found that only the H^+^ concentration has a significant effect on the gelation process of TEOS. The applied acids (HCl, HNO_3_, acetic acid) showed a volcanic curve with the maxima between pH = 1–2, almost indifferent to the type of acid. In their case, the ratio of water to TEOS was stoichiometric (water: TEOS = 4). However, there is no information on the synthesis of silica xerogels from TEOS using pTSA as the sole acid catalyst. The application of pTSA’s is mainly reported in post-synthesis surface functionalization of silica materials.

However, unmodified silicas typically exhibit low CO_2_ adsorption capacities, especially at ambient conditions, due to weak physisorptive interactions with CO_2_ molecules. To enhance the affinity of silica surfaces for CO_2_, researchers have introduced amine functional groups, which react with CO_2_ to form carbamate, carbamic acid, or bicarbonate species, depending on the type of amine and operating conditions [16,17]. Normally branched amines are preferred because they have a lower heat of adsorption than the primary amine, which makes the desorption of CO_2_ easier and less energy demanding. They also have a lower affinity for reacting with CO_2_ and forming urea which reduces the active sites of the material [18,19]. These amine-modified silicas are typically synthesized via grafting or impregnation of primary, secondary, or polyamines (e.g., polyethyleneimine (PEI), tetraethylenepentamine (TEPA), or 3-aminopropyltriethoxysilane (APTES)) onto the silica framework [20,21,22]. The presence of amines enables chemisorption of CO_2_, particularly at low partial pressures, and results in significantly improved capacity and selectivity over other gases like N_2_ [23].

A key advantage of mesoporous supports lies in their ability to accommodate high amine loadings without severely compromising pore accessibility, thus maintaining favorable adsorption–desorption kinetics [24]. Furthermore, the tunability of surface chemistry and pore architecture allows researchers to optimize the balance between amine content, CO_2_ capacity, and material regenerability [22]. Despite these advances, challenges remain, such as oxidative degradation of amines, pore blocking at high loadings, and stability during long-term cycling [25].

**In this work,** we present a novel synthetic strategy for the preparation of mesoporous silica xerogel without prehydrolysis of the silica in the precursor step. The method utilizes pTSA acid both as a drying control additive and as a porogenic agent. A systematic investigation was conducted to optimize the synthesis parameters and template removal process in order to obtain materials with superior structural and textural characteristics. Furthermore, the feasibility of recovering and reusing the porogenic agent was evaluated. The surface of the newly produced material was further modified with piperazine moieties by a two-step post-synthesis procedure. The obtained functionalized xerogel was studied for CO_2_ capture.

## 2. Experimental

### 2.1. Materials

Tetraethylorthosilicate (TEOS, Sigma Aldrich, St. Louis, MO, USA) was used as the silica source, *p*-toluenesulfonic acid (pTSA, monohydrate, 97%, Thermo Scientific Chemicals, Waltham, MA, USA) as porogen, and (3-chloropropyl)triethoxysilane (95%, Sigma Aldrich) and 1-(2-Hydroxyethyl) piperazine (99%, TCI) as modifying agents.

### 2.2. Synthesis of Mesoporous Silica

Briefly, after the complete dissolution of pTSA (molar ratios 1:1 and 1:3 TEOS:pTSA) in 50 mL of distilled water, TEOS was added to the solution dropwise. The resulting mixture was stirred for 120 min at room temperature, then dried at 30, 60, or 90 °C. The samples were named MSX-Y-Z, where MSX stands for mesoporous silica xerogel; Y = 30, 60, or 90 corresponding to the drying temperature in °C; and Z = 1 or 3 corresponding to the pTSA:TEOS molar ratio.

Removal of the porogen was investigated using two different methods: (i) high-temperature calcination and (ii) water extraction at room temperature. Calcination was performed at 550 °C for 5 h with a heating rate of 3 °C/min. Complete acid extraction was achieved through two cycles of vigorous stirring in water (1 g of silica–acid suspended in 100 mL of distilled water) for 1 h each.

The template-free samples were denoted as MSX-Y-Z-C (calcined) and MSX-Y-Z-W (washed). To investigate the mechanism of porous silica formation in more detail, a pTSA–silicate sample was also studied before template removal. Template-containing samples were marked with a “T” (MSX-Y-Z-T).

Additionally, a sample was prepared using recycled pTSA to evaluate the reusability of the acid in a second xerogel synthesis cycle. This sample was denoted as MSX-Y-Z-C-R.

### 2.3. Post-Synthesis Surface Modification

**Scheme 1:**
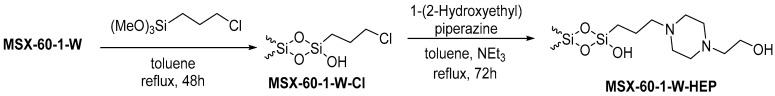
Preparation of HEP-modified MSX-60-1-W.

One gram of mesoporous silica (MSX-60-1-W) was suspended in 8.0 mL of dry toluene, and 1.0 g of (3-chloropropyl)trimethoxysilane was added. The mixture was refluxed for 48 h. The modified silica was filtered through a nylon membrane filter (pore size 0.45 μm, ø 47 mm) and washed with toluene to obtain the functionalized mesoporous silica, MSX-60-1-W-Cl (Scheme 1).

In the second step, 0.71 g of MSX-60-1-W-Cl was suspended in 7.0 mL of dry toluene containing 0.71 g of 1-(2-hydroxyethyl)piperazine, and the reaction mixture was refluxed for 72 h (Scheme 1). The solid phase was filtered through a nylon membrane filter (pore size 0.45 μm, ø 47 mm) and washed consecutively with toluene and ethanol. The resulting functionalized silica was denoted as MSX-60-1-W-HEP.

### 2.4. CO_2_ Adsorption Experiments

#### 2.4.1. CO_2_ Adsorption Measurements in Dynamic Conditions

CO_2_ adsorption experiments were performed under dynamic conditions using a flow-through system. Prior to adsorption, 0.40 g of adsorbent was dried at 150 °C for 2 h. The experiments were conducted using a gas mixture of 5 mL/min CO_2_ in N_2_ at a total flow rate of 30 mL/min. The gas composition was analyzed online using a NEXIS GC-2030 ATF gas chromatograph (Shimadzu, Tokyo, Japan) equipped with a 25 m PLOT Q capillary column.

CO_2_/water vapor co-adsorption experiments (3 vol.% CO_2_ and 1 vol.% water vapor) were also carried out under the same flow rate (30 mL/min). The amounts of adsorbed CO_2_ and water vapor were determined and used to calculate the adsorption capacities.

#### 2.4.2. CO_2_ Adsorption Measurements in Static Conditions

Static adsorption experiments were performed using an AUTOSORB iQ-MPAG instrument (Quantachrome Instruments, Anton Paar brand, Boynton Beach, FL, USA) with pure CO_2_ as the adsorbate at 0 °C and 25 °C. After evacuation, the sample holder vessel was filled with CO_2_ to a specific pressure. Once equilibrium was reached, the amount of CO_2_ adsorbed by the sample was determined. The adsorption isotherms were plotted as the equilibrium amount of CO_2_ adsorbed versus the relative pressure (*p/p_0_* = 0.001–0.03).

### 2.5. Characterization Techniques

High angle X-ray powder diffraction patterns were recorded using an X’Pert MD type (Malvern Panalytical Ltd., Almelo, The Netherlands) diffractometer applying monochromatized CuKα radiation (40 kV, 35 mA). Patterns were collected from 3 to 75°2θ, by 0.04° step size for 4 s.

The textural properties were determined by N_2_ physisorption at −196 °C using an AUTOSORB iQ-C-MP-AG-AG (Quantachrome Instruments, an Anton Paar brand, Ashland, VA, USA). Samples were pretreated at 150 °C under vacuum for 10 h. The specific surface area was calculated between relative pressures of 0.05 and 0.21 using the Brunauer–Emmett–Teller (BET) equation [21]. The total pore volume was estimated based on the amount of gas adsorbed at a relative pressure of *p/p_0_* = 0.98, according to Gurvich’s rule [22]. The Barrett–Joyner–Halenda (BJH) method was applied to evaluate the pore size distribution, and average pore size values were calculated from the desorption branch of the isotherm [23].

The NMR spectra were recorded on a Bruker Avance III HD 600 NMR spectrometer (Bruker, Karlsruhe, Germany) operating at 599.98 MHz ^1^H frequency (119.21 MHz for ^29^Si, 150.84 MHz for ^13^C)), using a 4 mm solid-state i-CP/MAS dual ^1^H/^31^P-^15^N probehead. The samples were packed in 4 mm rotors (Zr_2_O) and spun at a magic angle spinning (MAS) rate of 10 kHz in all experiments. The quantitative direct excitation ^29^Si NMR spectra were acquired with a single-pulse sequence, 90° pulse length of 2.3 s, time domain data points of 3 K, spectrum width of 71 kHz; 1024 transients were accumulated with a relaxation delay of 60 s between each scan. The spectra were zero-filled to 16 K data points and processed with an exponential window function (line broadening factor 20) before the Fourier transformation. The ^1^H-X (X = ^29^Si or ^13^C) cross-polarization MAS (CP MAS) spectra were acquired with the following experimental parameters: ^1^H excitation pulse of 2.5 µs, contact time of 5 ms for ^29^Si and 2 ms for ^13^C experiments and a relaxation delay of 5 ms. For the ^1^H-^29^Si CP spectra of the samples with removed pTSA 1024 scans were accumulated, while 3096 scans we used for the samples with pTSA. The number of scans for ^1^H-^13^C CPMAS spectra was 512. ^1^H SPINAL-64 decoupling scheme was used during acquisition of CP experiments. Octakis(trimethylsiloxy)silsesquioxane powder (Q8M8, δ^29^Si = 11.7 ppm) was used for ^29^Si chemical shift referencing as external standard. ^13^C chemical shifts were referenced to the carboxyl group peak of α-glycine (δ^13^C = 176.5 ppm).

Monitoring of residual organics and the efficiency of template removal was conducted by temperature-programmed oxidation–thermogravimetric analysis (TPO-TGA), performed using a STA449F5 Jupiter instrument (NETZSCH Gerätebau GmbH, Selb, Germany). Samples were placed in ceramic crucibles and heated in an air flow (50 cm^3^/min) at a rate of 10 °C/min up to 600 °C, followed by a final hold of 1 h.

## 3. Results and Discussion

### 3.1. Synthesis of Mesoporous Silica Xerogels at Different Synthesis Conditions and Composition

**Figure 1:**
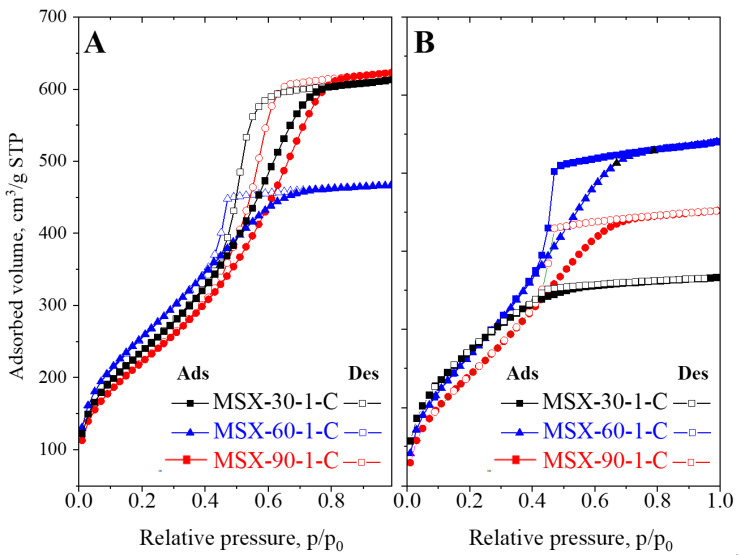
N_2_-physisorption isotherms of the samples dried at 30, 60, and 90 °C with TEOS-to-acid molar ratios 1:1 and calcined (**A**) or water extracted for template removal (**B**) (adsorption branch: filled symbols, desorption branch: empty symbols).

Porous silica xerogels were synthesized via a *p*-TSA-assisted sol–gel method. The synthesis parameters were systematically varied by applying different temperatures (30 °C, 60 °C, and 90 °C), *p*-TSA to tetraethyl orthosilicate (TEOS) molar ratios (1:1 and 3:1), and two different template removal strategies: calcination and extraction. The nitrogen adsorption–desorption isotherms, presented in Figure 1, can be categorized as type IV with an H2 hysteresis loop, indicative of ink-bottle-shaped mesopore formation [24]. The calculated textural parameters are summarized in Table 1. The strong Brønsted acidity of *p*-TSA, bearing a sulfonic acid functional group (–SO_3_H), enables it to donate protons (H^+^) and subsequently acquire a negative charge. This facilitates electrostatic interactions with positively charged silane species during the sol–gel process, thereby playing a pivotal role in directing the structural evolution of the silica xerogels. The synthesis was carried out in a highly diluted medium (H_2_O:TEOS = 138), where the condensation process is very slow and proceed predominantly during the subsequent drying and calcination step. Takahashi et al. [26] systematically investigated the influence of citric acid on silica xerogel formation and they found that both the solubility of the organic acid and its relatively weak interaction with surface silanols are critical parameters governing mesopore development. Acids with high aqueous solubility are capable of swelling the silica gel network, thereby mitigating pore shrinkage during the drying process. Furthermore, weak silanol–acid interactions modulate the assembly of the silica framework without significantly impeding the condensation of silica oligomers. Consequently, a rigid, highly porous gel architecture is obtained. p-toluenesulfonic acid (pTSA) exhibits a comparably higher solubility in comparison to citric acid (pTSA: 67 g/100 mL; CA: 58 g/100 mL in water), but its acidity is much stronger (pTSA: pKa_1_ = –1.3; CA: pKa_1_ = 3.2). The pronounced acidity of the synthesis medium (pH < 1) promoted extensive hydrolysis of TEOS, facilitating the formation of mono- and oligomeric silica species that subsequently assembled into a polymeric network. The strong interaction between *p*-TSA and the silica matrix is further evidenced by thermogravimetric analysis (Figure S1), which shows that the thermal decomposition of the acid in the silica–acid composite occurs at higher temperatures compared to pure *p*-TSA (melting point: 105–107 °C for the monohydrate [25]). Moreover, the synthesis was conducted without a pre-hydrolysis step of the TEOS precursor, allowing the intrinsic acidity of *p*-TSA to directly influence the hydrolysis–condensation dynamics. Such strong interactions affect the condensation pathway, leading to a less rigid silica framework and the development of smaller mesopores relative to citric acid. In this context, the role of pTSA extends beyond simple drying control, with its templating effect becoming the dominant factor. This templating influence is further reinforced by the hydrate-forming tendency and hygroscopic nature of pTSA, which facilitate the formation of water-rich domains during drying, likely giving rise to larger nanocrystalline structures within the gel matrix.

The observed hysteresis loop morphology is consistent with the generation of narrow pore entrances in an ink-bottle-type pore system, likely resulting from partial shrinkage of the silica framework during drying and calcination. The drying temperature plays a critical role, influencing both the crystallization behavior of the acid template and the kinetics of silica precursor condensation. Drying at three distinct temperatures (30 °C, 60 °C, and 90 °C) led to variations in the textural properties of the synthesized materials (Figure 1A). Among these, 60 °C was identified as the optimal temperature for the formation of xerogels with desirable textural properties and reasonable energy demand (Table 1). For comparison, we used nitric acid, with the same pKa1 value like pTSA (~−1.3) using the same synthesis parameters, and a microporous xerogel was formed containing mainly supermicropores (0.7–2 nm), as could be expected in acidic media (Figure S2).

For the sample prepared with a TEOS:acid molar ratio of 1:3 (MSX-60-3-C), the adsorption–desorption isotherm retained the H2-type hysteresis loop (Figure 1B), although a noticeable shift toward higher relative pressures was observed. A higher amount of *p*-TSA in the synthesis mixture also resulted in a decreased specific surface area. This shift, accompanied by an increase in total pore volume, suggests the formation of pores with larger diameters.

To evaluate the impact of the acid removal procedure on the silica properties, the sample prepared at 30, 60, and 90 °C with a TEOS:acid ratio of 1:1 was subjected to either high-temperature calcination or room-temperature water extraction. The resulting isotherms (Figure 1B) exhibited no substantial differences in type or shape; however, the specific surface area and total pore volume (Table 1) of the water-extracted sample were slightly higher than those of the washed ones. Probably the most soft procedure for acid removal preserved the surface area of the obtained silica xerogels.

This improvement in textural characteristics can be attributed to the milder conditions of the extraction process, which likely minimize structural collapse and prevent pore shrinkage, thereby preserving the porous network more effectively. These findings indicate that both the acidity and concentration of the porogenic agent, as well as the template removal procedure, play critical roles in modulating the textural characteristics of the mesoporous silica framework.

The crystalline phases appearing in the silica-acid composites after drying at different temperatures were studied by X-ray powder diffraction. In the low-angle XRD patterns of the silica-organic acid composites, characteristic reflections of ordered pore structure cannot be observed. In high-angle patterns (Figure S3), the crystalline forms of pTSA (ICDD card No. 41-1636) can be witnessed besides the amorphous halo of silica at around 25°2*θ*. pTSA preserves its original hydrate form by mixing with TEOS; however, the samples show a hygroscopic nature. The presence of crystalline pTSA deposited on the external surface of the silica materials indicates a hindering of the formation of textural mesoporosity.

In pursuit of a sustainable, environmentally friendly, and energy-efficient approach for obtaining mesoporous silicates, the pores of silica–organic acid composites were freed by two different techniques: calcination and extraction. The conventional method for organic matter removal, calcination, was implemented at 550 °C for 5 h with a heating rate of 3 °C/min. Notably, the extraction technique permits the regeneration and subsequent reuse of the organic acids, whereas these compounds are entirely destroyed during calcination.

Materials derived via the extraction method exhibited higher surface area and pore volume compared to calcined ones (Table 1 and Figure 1), suggesting the potential to supplant the energy-demanding calcination process utilizing a non-toxic solvent. The applied temperature of calcination leads to the shrinkage of the silica network and dehydroxylation of the silanol groups on the surface, as was confirmed by NMR (Table 2), resulting in the formation of a more condensed silica matrix with a lower amount of surface groups and lower surface area. The organics content of the washed samples was checked by TG analysis, and the weight loss corresponded only to dihydroxylation process of silica over 500 °C. By the applied simple washing procedure, the acid could be totally removed, collected, and reused. Moreover, the lack of a high-temperature calcination step allows for the mesoporous silica to retain more silanol groups [27].

**Figure 2:**
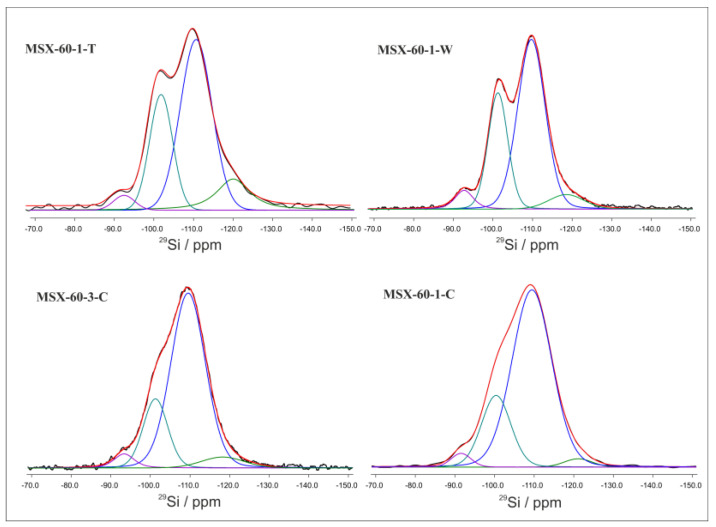
Direct excitation ^29^Si NMR spectra of the studied materials.

**Figure 3:**
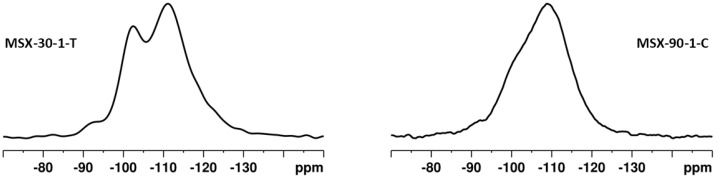
Direct excitation ^29^Si NMR spectra of MSX-30-1-T and MSX-90-1-C materials.

Solid state NMR spectroscopy was used to obtain a more detailed insight into the effect of the synthesis conditions on structural characteristics of the obtained mesoporous silica xerogels. Direct excitation ^29^Si and ^1^H→^29^Si CPMAS spectra were used to investigate the structural characteristics of the silica matrix in the presence of the pTSA as well as after the template removal. Chemical shifts and relative intensities of the resonances in the direct excitation ^29^Si NMR spectra provide information about the type and quantitative distribution of the different (SiO)^n^ Si(OH)_4-n_ species (n = 1, 2, 3; e.g., Q^n^ species) of the silica matrix. The ^29^Si NMR spectra of the MSX materials display similar spectral pattern consisting of four partially overlapping resonances. Figure 2 presents the direct excitation ^29^Si spectra of acid containing MSX prepared at 60 °C (MSX-60-1-T), calcined MSX samples prepared at 60 °C with an acid:TEOS ratio 1 or 3 (MSX-60-1-C, MSX-60-3-C) and washed MSX-60-1-W materials. The spectra show a pair of resonances in the spectral region between −110 to −120 ppm that is typical for the Q^4^ species [Si(0OH) structural units] representing the main building blocks of the bulk silicate framework. The typical chemical shift value for the Q^4^ species in the silica materials is usually at around −111 ppm. The observation of the second high field resonance at −118 ppm indicates structural diversity and the presence of Q^4^ species with distorted bond length and valence angles due to the inclusion of the template. We suggest that this resonance originates mainly from Q^4^ species in close proximity to the organic acid molecules, since its intensity decreases significantly after removal of the template. We conjecture that the second types of Q^4^ species do not have a significant impact on material properties since their quantity after the template removal is minor (2 ÷ 6%) and practically identical in most materials without the template. The resonance at around −101 ppm is characteristic for Q^3^ structural species [Si(1OH) units], while the low intensity signal at around −92 ppm indicates the presence of a small amount of Q^2^ structures [Si(2OH) units]. The ^29^Si spectra of samples MSX-30-1-T and MSX-90-1-C show similar spectral characteristics (Figure 3).

The Q^3^ and Q^2^ species originate from defect sites at silica surface and/or at pore edges. The quantitative distributions of the different types of structures were calculated by deconvolution of the spectral patterns in the ^29^Si spectra and are summarized in Table 2. The analysis of the data shows that Q^4^ was between 66 and 77% while the fraction of the Q^3^ + Q^2^ species was between 34 and 23%. The formation of a negligible amount of Q^1^ (Q^1^ = Si(3OH)) species was detected in MSX-30-1-T. The analysis of the direct excitation ^29^Si spectra of the samples where the template was removed by calcination at 550 °C indicates that calcination resulted in some structural changes of the silica framework. In all calcined samples the amount of Q^4^ units increased, from 70 up to 77% at the expense of the silanol groups (Q^3^ + Q^2^ species decreasing from 30 down to 23%), Table 2. The decrease in the fraction of Q^3^ and Q^2^ species and the increase in the fraction of Q^4^ structural units after calcination suggest that the heating resulted in consolidation of the silica framework via thermal condensation of the silanol groups, thus increasing the amount of the highly cross-linked Q^4^ species. The MSX-60-1-W sample shows the presence of a higher amount of Q^3^ + Q^2^ (34%) and the lowest content of Q^4^ in comparison to its calcined analogs.

**Figure 4:**
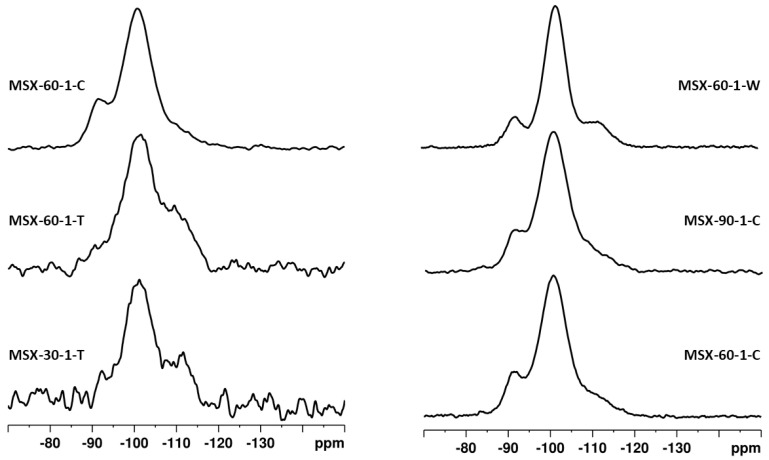
^1^H-^29^Si CPMAS spectra of the studied materials.

**Figure 5:**
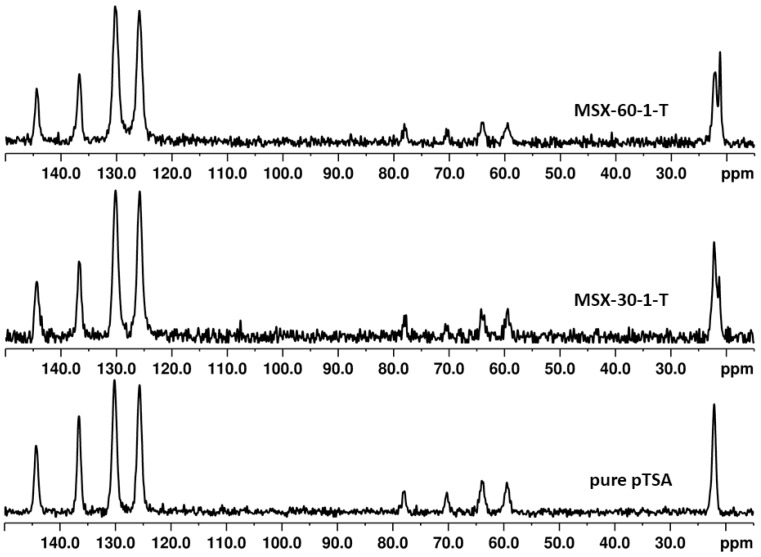
^1^H-^13^C CP MAS spectra of the pure pTSA and the silica xerogels with pTSA. (The low intensity signals in the region from 80 to 55 ppm are spinning side bands of the aromatic resonances resulting from sample rotation at the magic angle).

Additional characterization of the studied materials was performed by ^1^H-^29^Si CPMAS spectra where the resonances of the silanol groups are specifically enhanced. The spectra are presented in Figure 4. The spectra of the samples containing the pTSA (MSX-30-1-T and MSX-60-1-T) have poor signal to noise ratio, indicating that transfer of magnetization from the protons to the neighboring Si centers is not efficient, resulting in insufficient Si-signal enhancement. The possible explanation for this could be the high mobility of the small organic molecules in the silica xerogels. In the ^1^H-^29^Si CPMAS spectra of materials obtained after removal of the template molecules, the resonances of the silanol groups from Q^3^ and Q^2^ structural units are significantly enhanced while the signal for the Q^4^ structures has the lowest intensity due to the lack of protons in close proximity. Finally, to investigate the possible changes of the porogen pTSA molecules during the preparation procedure, we measured ^1^H-^13^C CPMAS spectra of the pure crystalline pTSA and of the silica xerogels with pTSA before its removal (Figure 5). The spectra show the four characteristic resonances in the aromatic region and the signal of the CH_3_ group at 22 ppm. The narrow signal line shapes indicate that pTSA is in a crystalline state in all samples. The additional resonance at 21.3 ppm detected in the spectra of MSX-30-1-T and MSX-60-1-T could be assigned to toluene, which is a product of pTSA hydrolysis taking place in the water solution during preparation of the xerogels.

**Figure 6:**
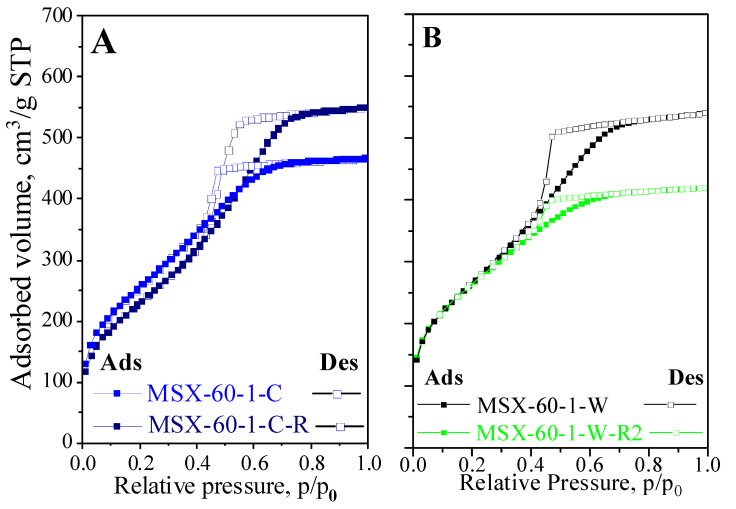
N_2_-physisorption isotherms of silica xerogels prepared with fresh and recycled pTSA acid (TEOS-to-acid molar ratio of 1:1), dried at 60 °C and calcined (**A**) and dried at 60 °C and washed (**B**) (adsorption branch: filled symbols, desorption branch: hollowed symbols).

To recycle the extracted acid, the filtrate was collected and recrystallized at 60 °C. The yield of the recovered pTSA was in the interval of 95–99%. The pTSA obtained by extraction from the MSX-60-1-T sample was characterized by the X-ray powder diffraction (XRD). The crystallinity and the structure of the recrystallized pTSA was found to be identical to the pristine compound, confirming the successful recovery of the acid. The recovered pTSA from the MSX-60-1-T samples was subsequently employed in three synthesis cycles of the MSX-60-1-C and MSX-60-1-W xerogels. The corresponding nitrogen isotherms of the initial and the third samples prepared with recovered acid are shown in Figure 6.

The silica xerogels synthesized using the recycled pTSA exhibited comparable physicochemical properties to those obtained with freshly used porogen. The average values with standard deviations for the textural parameters of the MSX-60-1-C and the MSX-60-1-W samples prepared with recovered pTSA are summarized in Table 3.

### 3.2. Surface Modification of the Porous Silica

The water-extracted silica xerogel, synthesized at 60 °C with TEOS-to-acid ratio of 1:1 and washed for acid removal, was selected for further modification with HEP (Scheme 1) because of its appropriate textural properties and optimal energy consumption. TG curves revealed 27 wt. % organic functional group content on the MSX-60-1-W sample.

The modification with HEP resulted in a significant surface decrease and partial pore blocking (Figure S4).

**Figure 7:**
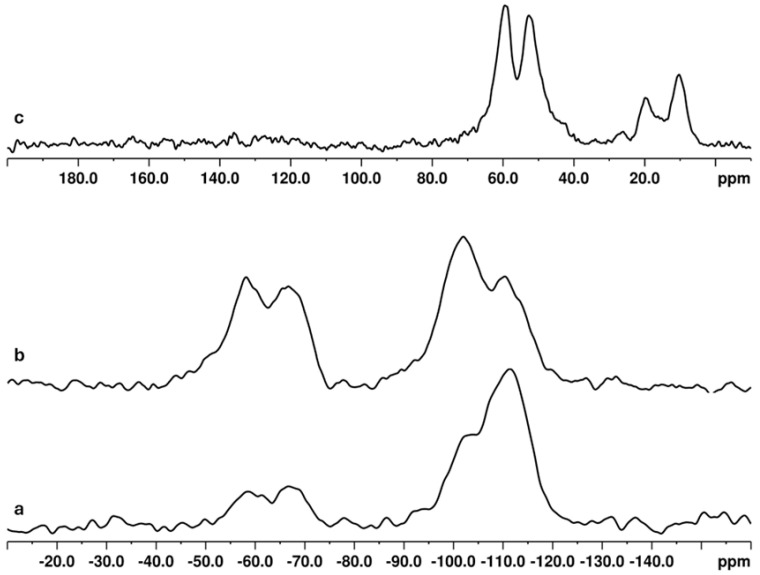
Solid state NMR spectra of MSX-60-1-W-HEP: (**a**) quantitative direct excitation ^29^Si spectrum, (**b**) ^1^H-^29^Si CPMAS spectrum, (**c**) ^1^H-^13^C CPMAS.

The modification with HEP linker was further confirmed by direct excitation ^29^Si NMR spectra, as well as by ^1^H-^29^Si CPMAS and ^1^H-^13^C CP NMR spectra (Figure 7). The quantitative assessment of functionalization was obtained by integration of the spectral patterns in the direct excitation ^29^Si spectra (Figure 7a), showing a functionalization degree of 22% that correlates well with the one determined by thermogravimetric analysis. The presence of the resonances at around −59 and −67 ppm in the ^29^Si and ^1^H-^29^Si CP (Figure 7b) spectra typical for the T^2^ [(SiO)_2_Si-(R1)-OR2)] and T^3^ [(SiO)_3_Si-R1] structural units (R1 = HEP; R2 = CH_2_CH_3_) from the silica framework additionally confirms the successful functionalization with organic groups. In the ^1^H-^29^Si CP spectrum, the resonances of the T^2^, T^3^, and Q^3^ structural units are selectively enhanced due to transfer of magnetization to Si centers from the neighboring protons from the organic moieties and the silanol groups. In addition, the ^1^H-^13^C CP spectrum of sample MSX-60-1-W-HEP shows characteristic resonances for the methylene groups of the different structural fragments of HEP (Figure 7c).

### 3.3. Adsorption Capacity and Selectivity for CO_2_ Adsorption

**Figure 8:**
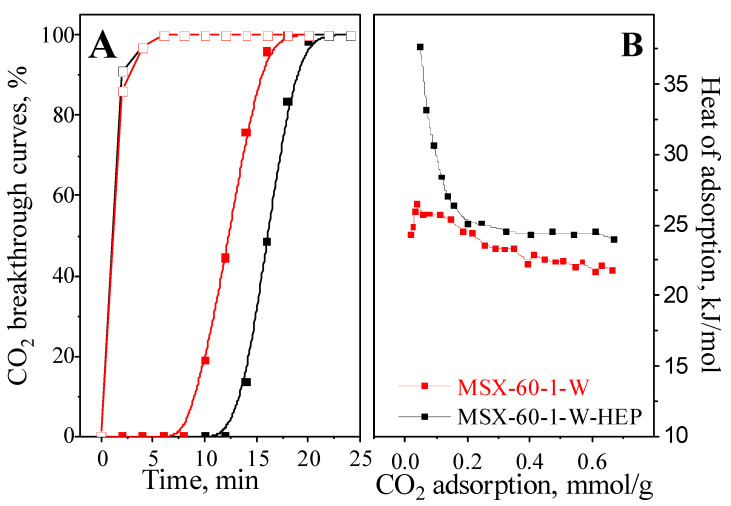
N_2_/CO_2_ breakthrough curves at dynamic conditions (5 mL/min CO_2_ in N_2_ at a total flow rate of 30 mL/min) (hollowed symbols are for N_2_ adsorption and filled symbols are for CO_2_ adsorption) (**A**) and heat of adsorption curves for the studied samples (**B**).

Breakthrough curves for CO_2_ adsorption in dynamic conditions with 5% CO_2_/40%N_2_ flow are shown in Figure 8. It was found that the functionalized mesoporous silica materials adsorbed a higher amount of CO_2_ than the initial one (Table 4). The main adsorption sites in MSX-60-1-W are silanol groups, which limits the adsorption behavior.

The MSX-60-1-W-HEP material shows about 30% higher CO_2_ capture capacity (4.4 mmol/g) compared to the parent silica xerogel (3.4 mmol/g) (Table 4). The HEP functionalization resulted in a higher content of adsorption sites with stronger basic properties in comparison to the parent mesoporous xerogel. Moreover, the modified material shows higher selectivity to CO_2_ over N_2_, based on IAST theory (Table 4).

The comparison with other similar amine-modified mesoporous silica materials is presented in Table 5. Our material showed the highest CO_2_ capacity in comparison to the other studied samples. Moreover, the CO_2_ capacity of MSX-60-1-W-HEP, which is modified with the much cheaper 1-(2-hydroxyethyl)piperazine is higher in comparison to our previously obtained MCM-48-P, which was modified with more expensive 1-methylpiperazine [28].

Total CO_2_ desorption could be registered at 60 °C for the functionalized silica sample. Leaching of the active sites was not observed after the adsorption experiments by TG analysis. The CO_2_ capacity was not decreased significantly after ten consecutive adsorption–desorption cycles (Table 2). The selectivity for CO_2_ adsorption was tested in the presence of 1 vol.% water vapor at a flow rate of 45 mL/min (CO_2_/H_2_O/N_2_). A small increase of the adsorption capacities (4.5 mmol/g) was registered in the presence of water vapor compared to dry gas mixture (CO_2_/N_2_). Most probably, the chemisorption of CO_2_ is responsible for higher selectivity (Table 2), as it was also detected in our previous paper [28]. Isosteric heat of adsorption for CO_2_ was calculated from the adsorption isotherms by using the Clausius–Clapeyron equation, and the results are presented in Figure 4. The results show that both parent and HEP-functionalized MSX-60-1-W samples provided effective weak adsorption sites for CO_2_ with a heat of adsorption value between 25–38 kJ/mol. The functionalized silica material exhibited increased heat of adsorption at low adsorbed amounts, probably due to the chemisorption of CO_2_ on functional groups at low coverage of the surface [32]. The modification of silica with HEP resulted in enhanced isosteric heat of adsorption due to the stronger interaction between functional groups and CO_2_ molecules. Therefore, HEP groups could be considered as more effective adsorption sites than silanol groups.

## 4. Conclusions

A pTSA-assisted procedure was used for the successful synthesis of porous silica xerogels under various synthesis conditions. Key variables included the concentration of acid, drying temperature, and the method of acid removal. The results demonstrated that both the drying temperature and the amount of pTSA significantly influenced the surface area, pore volume, and pore structure. The use of pTSA as a template led to the formation of silica with a high surface area and pore volume. Environmentally friendly template removal via water extraction produced materials with superior textural properties compared to traditional calcination, while enabling the recovery and reuse of pTSA with over 95% efficiency.

Synthesis at 60 °C using a TEOS-to-acid ratio of 1:1 and water extraction for acid removal resulted in optimal textural and structural characteristics. The 1-(2-hydroxyethyl)piperazine-modified mesoporous MSX-60-1-W xerogel was successfully synthesized via a simple two-step post-synthesis procedure. A higher CO_2_ adsorption capacity was observed for the 1-(2-hydroxyethyl)piperazine-functionalized xerogel (4.4 mmol/g), compared to the non-modified silica (3.4 mmol/g). Modification with 1-(2-hydroxyethyl)piperazine resulted in a higher isosteric heat of adsorption than that of the pure silica material, due to stronger interactions between the surface functional groups and CO_2_ molecules. Complete CO_2_ desorption from the functionalized material was achieved at 60 °C, with no leaching of functional groups detected after four consecutive adsorption cycles.

## Figures and Tables

**Table 1:** Textural properties of porous silica xerogels.

Samples	Specific Surface Area m^2^/g	Total Pore Volume cm^3^/g
MSX-60-1-C	885	0.91
MSX-60-3-C	796	0.94
MSX-30-1-C	868	0.95
MSX-90-1-C	817	0.97
MSX-60-1-W	980	0.84
MSX-30-1-W	990	0.57
MSX-90-1-W	886	0.70

**Table 2:** ^29^Si NMR data of as-prepared samples containing organic acids and their calcined analogues. ^a^ Q4 silicon signal at −111 ppm, ^b^ Q4 silicon signal at −122 ppm.

Samples	Q_1_/Si(3OH)%	Q_2_/Si(2OH)%	Q_3_/Si(1OH)%	Q_4_/Si(0OH) ^a^ %	Q_4_/Si(0OH) ^b^ %
MSX-30-1-T	1	5	24	54	16
MSX-60-1-T	-	3	27	56	14
MSX-60-1-C	-	3	22	73	2
MSX-60-3-C	-	4	20	71	5
MSX-90-1-C	-	3	20	72	5
MSX-60-1-W	-	5	29	60	6

**Table 3:** Textural properties of porous silica samples.

Samples	Specific Surface Area, m^2^/g	Total Pore Volume, cm^3^/g
MSX-60-1-C	885	0.91
MSX-60-1-C-R	860 ± 18	0.85 ± 0.14
MSX-60-1-W	980	0.84
MSX-60-1-W-R	970 ± 15	0.65 ± 0.10

**Table 4:** CO_2_ adsorption capacities of the prepared materials in dynamic conditions and heat of adsorption.^1^ 5 mL/min CO_2_ in N_2_ at a total flow rate of 30 mL/min; ^2^ 3 vol.% CO_2_ and 1 vol.% water vapor at a flow rate (30 mL/min, ^3^ in ten consecutive adsorption-desorption cycles.

Samples	CO_2_ Adsorption from CO_2_/N_2_^1^, mmol/g	Selectivity of CO_2_ Over N_2_, Based on IAST Theory	Adsorption of CO_2_ from CO_2_/H_2_O/N_2_^2^, mmol/g
MSX-60-1-W	3.4 (3.2) ^3^	272	3.3
MSX-60-1-W-HEP	4.4 (4.1) ^3^	352	4.5

**Table 5:** Comparison of the CO_2_ adsorption capacity from CO_2_/N_2_ on the amine modified mesoporous silica materials.

Samples	Modification Groups	CO_2_ Adsorption from CO_2_/N_2_, mmol/g	References
MSX-60-1-W-HEP	1-(2-hydroxyethyl)piperazine	4.4	This work
SBA-15/DAPTES	DAPTES	3.9	[29]
MCM-48-P	1-methylpiperazine	4.4	[28]
MCM-41-PEI 50	Polyethyleneimine	2.5	[30]
SBA-15-PEI 50	Polyethyleneimine	2.9	[30]
SBA-16-PEI 50	Polyethyleneimine	2.9	[30]
MCM-48-PEI 50	Polyethyleneimine	2.7	[30]
KIT-6-PEI 50	Polyethyleneimine	3.1	[30]
25MSU-J-TEPA-20	tetraethylenepentamine	3.2	[31]
45MSU-J-TEPA-50	tetraethylenepentamine	3.1	[31]
45MSU-J-TEPA-70	tetraethylenepentamine	3.5	[31]
65MSU-J-TEPA-50	tetraethylenepentamine	3.7	[31]

## Data Availability

Data is contained within the article or Supplementary Material.

## Supplementary Materials

The following supporting information can be downloaded at: https://www.mdpi.com/article/10.3390/nano15191459/s1, Figure S1. TG curve profile for weight loss of sample MSX-60-1-T (silica-template composite); Figure S2. N2-physisorption isotherms of the MS-HNO3 sample dried at 60 °C; Figure S3. XRD patterns of samples MSX-60-1-T and MSX-30-1-T (silica-template composite); Figure S4. The pore size distribution of MSX-60-1-W-HEP.
