# Supplementary material for: Mesoporous Silica Xerogels Prepared by p-toluenesulfonic Acid-Assisted Synthesis: Piperazine-Modification and CO2 Adsorption
Source: Nanomaterials (Basel). 2025 Sep 23;15(19):1459. doi: 10.3390/nano15191459 (PMC12525869; doi:10.3390/nano15191459)
Supplement: Supplementary file 1 [file nanomaterials-15-01459-s001.zip › nanomaterials-3849700-supplementary.pdf]

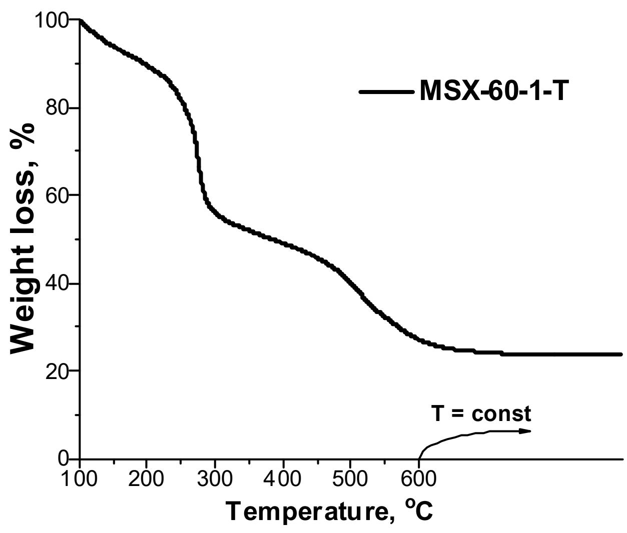

Figure S1. TG curve profile for weight loss of sample MSX-60-1-T (silica-template composite)

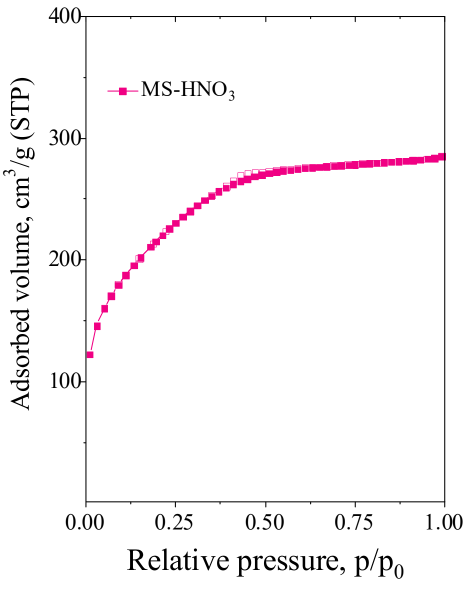

Figure S2. N_2-physisorption isotherms of the MS-HNO_3 sample dried at 60°C

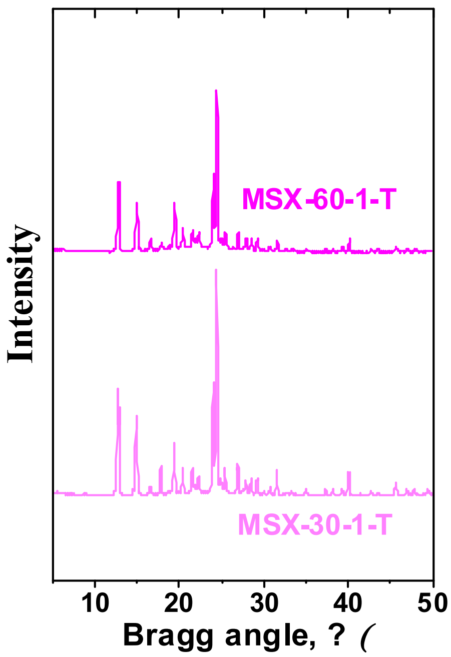

Figure S3. XRD patterns of samples MSX-60-1-T and MSX-30-1-T (silica-template composite)

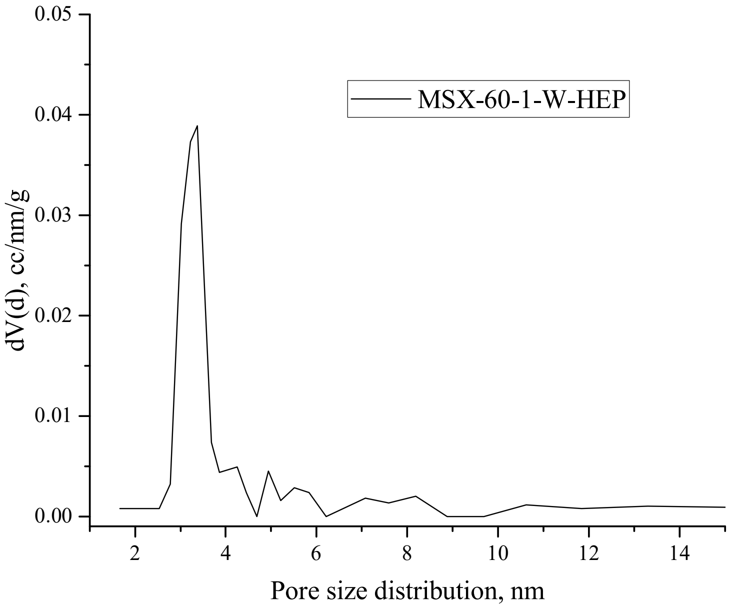

Figure S4. The pore size distribution of MSX-60-1-W-HEP.
